# Non‐digestible carbohydrates supplementation increases *miR‐32* expression in the healthy human colorectal epithelium: A randomized controlled trial

**DOI:** 10.1002/mc.22666

**Published:** 2017-05-09

**Authors:** Fiona C Malcomson, Naomi D Willis, Iain McCallum, Long Xie, Bart Lagerwaard, Seamus Kelly, D. Michael Bradburn, Nigel J Belshaw, Ian T Johnson, John C Mathers

**Affiliations:** ^1^ Human Nutrition Research Centre, Institute of Cellular Medicine, Newcastle University Campus for Ageing and Vitality Newcastle upon Tyne UK; ^2^ Northumbria Healthcare NHS Foundation Trust North Shields UK; ^3^ Northumbria Healthcare NHS Foundation Trust Ashington UK; ^4^ Institute of Food Research Norwich Research Park Norwich Norfolk UK

**Keywords:** colorectal cancer, microRNA, non‐digestible carbohydrates

## Abstract

Colorectal cancer (CRC) risk is modulated by diet and there is convincing evidence of reduced risk with higher non‐digestible carbohydrates (NDCs) consumption. Resistant starch (RS), a NDC, positively modulates the expression of oncogenic microRNAs, suggesting that this could be a mechanism through which NDCs protect against CRC. The present study aimed to investigate the effects of supplementation with two NDCs, RS, and polydextrose (PD), on microRNA expression in the macroscopically‐normal human rectal epithelium using samples from the DISC Study, a randomized, double‐blind, placebo‐controlled dietary intervention. We screened 1008 miRNAs in pooled post‐intervention rectal mucosal samples from participants allocated to the double placebo group and those supplemented with both RS and PD. A total of 111 miRNAs were up‐ or down‐regulated by at least twofold in the RS + PD group compared with the control group. From these, eight were selected for quantification in individual participant samples by qPCR, and fold‐change direction was consistent with the array for seven miRNAs. The inconsistency for *miR‐133b* and the lower fold‐change values observed for the seven miRNAs is probably because qPCR of individual participant samples is a more robust and sensitive method of quantification than the array. *miR‐32* expression was increased by approximately threefold (*P* = 0.033) in the rectal mucosa of participants supplemented with RS + PD compared with placebo. *miR‐32* is involved in the regulation of processes such as cell proliferation that are dysregulated in CRC. Furthermore, *miR‐32* may affect non‐canonical NF‐κB signaling via regulation of *TRAF3* expression and consequently *NIK* stabilization.

**Abbreviations**
CRC: colorectal cancer
HDAC: histone deacetylase
miRNA: microRNA
NDC: non‐digestible carbohydrates
PD: polydextrose
RS: resistant starch
SCFA: short chain fatty acid

## 1 INTRODUCTION

Colorectal cancer (CRC) is the third most common cancer worldwide and fourth most common cause of cancer‐related death.^1^ CRC risk is significantly modulated by lifestyle factors including diet. The World Cancer Research Fund/American Institute for Cancer report concluded that there is convincing evidence for lower CRC risk with higher consumption of dietary fibre.^2^ This includes non‐digestible carbohydrates (NDCs), such as resistant starch (RS) and polydextrose (PD), that resist digestion in the small intestine and are fermented by bacteria in the large bowel to produce the short‐chain fatty acids (SCFAs) butyrate (the preferred energy source for colonocytes), propionate and acetate.

Butyrate, produced primarily by bacterial species of the *Clostridia, Roseburia*, and *Eubacteria* genera,^3^ has chemoprotective anti‐inflammatory and anti‐proliferative properties and regulates apoptosis in both normal and cancerous cells.^4^, ^5^ Butyrate has effects on epigenetic mechanisms that regulate gene expression and are important in colorectal tumorigenesis, including its role as a histone deacetylase inhibitor (HDACi)^6^ and effects on microRNA (miRNA) expression.^7^, ^8^

miRNAs are small, non‐coding RNAs that regulate the expression of their target genes at a post‐transcriptional level by degrading messenger RNA or blocking translation. Over 1500 miRNAs have been identified in humans and have been predicted to regulate the expression of up to 60% of genes,^9^, ^10^ consequently regulating multiple biological processes including cell proliferation, differentiation, and apoptosis. Abnormal miRNA expression is observed in colorectal tissue as well as plasma, urine, and stool samples from CRC patients.^11^, ^12^

miRNA expression is modulated by multiple dietary factors; for example the expression of 250, 198, and 99 miRNAs was associated with intakes of carbohydrate, sucrose, and wholegrains, respectively, and some of these miRNAs showed differential expression between normal and tumor tissue.^13^ A limited number of studies have investigated the effects of NDCs and butyrate on miRNA expression in the cells from the large bowel epithelium, and the majority of studies investigating the role of miRNAs in colorectal carcinogenesis have been performed in CRC cell lines or using tissue from resected tumors. Using a microarray‐based approach, Humphreys and colleagues observed that treatment of HT‐29 colorectal adenocarcinoma cells with 5 mM butyrate for 48 h altered the expression 69 miRNAs.^14^ Follow‐up analysis by qPCR showed that butyrate treatment reduced expression of miRNAs belonging to the oncogenic *miR‐17‐92* cluster and decreased cell proliferation. Hu et al also reported that butyrate exposure altered expression of 44 miRNAs in HCT‐116 cells, 18 of which were confirmed by qPCR.^7^ Humphreys et al were the first to investigate the effects of RS and butyrate on expression of *miR‐17‐92* cluster members in a human intervention study.^15^ Supplementation of a high red meat diet, which increased *miR‐17‐92* expression by approximately 30%, with butyrylated RS for 4 wk restored expression to baseline levels, suggesting that RS reversed the detrimental effects of red meat on expression of oncogenic miRNAs.

In the current study, we hypothesised that NDCs are protective against CRC by regulating the expression of miRNAs, in particular by down‐regulating expression of miRNAs that are upregulated in CRC. For the first time, we used a whole miRNome PCR array to screen for candidate miRNAs that are differentially expressed in the macroscopically‐normal rectal mucosa of healthy participants following supplementation with two NDCs (RS and PD). We validated the findings from the miRNome array for eight miRNAs by qPCR using RNA samples from 29 individual participants.

## 2 METHODS

### 2.1 Study participants

Participants were recruited from gastroenterology out‐patients departments at North Tyneside General Hospital, North Shields, UK and Wansbeck General Hospital, Ashington, UK between May 2010 and July 2011. The exclusion criteria included being aged <16 or >85 years, being a prisoner at the time of endoscopy, being pregnant, or planning to become pregnant, diabetes mellitus, familial adenomatous polyposis syndrome, Lynch Syndrome, known colorectal tumor or prior colorectal cancer, prior colorectal resection, active colonic inflammation at endoscopy, iatrogenic perforation at endoscopy, incomplete left‐sided examination, colorectal carcinoma discovered at endoscopy, CRC on histology, chemotherapy in the last 6 months, administering non‐steroidal anti‐inflammatories, anti‐coagulants, or immunosuppressive medication.

### 2.2 Intervention methodology

The samples utilized for the present study were collected as part of the DISC Study, a double‐blind, randomized, placebo‐controlled dietary intervention that supplemented healthy participants with RS and/or PD for 50 d in a 2 × 2 factorial design.^16^ For the purpose of this study, the following two groups of participants were used:

- Double placebo (Control): 12 g of Maltodextrin and 23 g of Amioca starch.
- Double intervention (RS + PD): 23 g of Hi‐maize® 260 and 12 g of Litesse® *Ultra*™. For the purposes of this paper, this group is described as the Intervention group.

Anthropometric measurements were taken at baseline and post‐intervention. Participants also completed a food frequency questionnaire and a lifestyle questionnaire that collected information on physical activity and smoking status.

### 2.3 Human colorectal tissue samples

This study was approved by the Newcastle and North Tyneside Research Ethics Committee (REC No. 09/H0907/77). Mucosal biopsies from the rectum (10 cm from the ano‐rectal verge) were collected pre‐intervention at endoscopy (colonoscopy or flexible sigmoidoscopy) and post‐intervention at rigid sigmoidoscopy.

### 2.4 Study design

**Figure 1:**
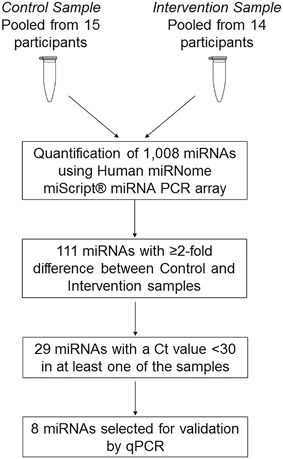
Flow‐chart showing the study design and selection of miRNAs for quantification in individual samples by qPCR. From 1008 miRNAs quantified, 111 miRNAs showed a difference in expression between pooled control and intervention samples. From these, eight miRNAs were selected for quantification in individual participant samples

We designed a two‐phase study to identify miRNAs modulated by NDCs (see Fig. 1). In the screening phase, we pooled equivalent amounts of cDNA reverse‐transcribed from RNA extracted from the rectal mucosa of each of 15 Control participants (supplemented with double placebo) and of 14 Intervention participants (supplemented with RS and PD). Abundance of 1008 miRNAs was quantified in each pooled sample by qPCR using the Human miRNome miScript® miRNA PCR array (Qiagen). A total of 111 miRNAs were up‐ or down‐regulated by more than twofold in the Intervention compared with the Control sample. This number was reduced to 29 miRNAs by excluding those which did not have a Ct value of <30 in at least one of the samples. From these, a total of seven miRNAs were selected to be validated by qPCR in the individual pre‐ and post‐intervention rectal mucosa samples. In addition, *miR‐32* was selected for quantification by qPCR due to its role in colorectal carcinogenesis.^17^

### 2.5 Isolation of total RNA

Total RNA was extracted from half a rectal mucosal biopsy using the miRNeasy Mini Kit (Qiagen, UK) following the manufacturer's instructions. Tissue disruption was performed by shaking the tissues with five 3 mm glass beads (VWR, UK) for 1 m in QIAzol Lysis Reagent (miRNeasy Mini Kit) using an amalgamator. The lysate and beads were transferred to QiaShredders (Qiagen, UK) for homogenization by centrifugation for 2 m at maximum speed. RNA was eluted in 30 µL of RNase‐free water. Concentration and RNA purity were determined using the NanoDrop 1000 spectrophotometer (Thermo Scientific) and NanoDrop 1000 Software version 3.7.1. RNA integrity was assessed by agarose gel electrophoresis.

### 2.6 Synthesis of cDNA

cDNA was synthesized from 0.8 μg of RNA using the miScript II RT Kit (Qiagen) as described by the manufacturer. The miScript HiSpec Buffer (5×) was used during reverse transcription to enable subsequent quantification of mature miRNAs. The samples were incubated for 60 m at 37°C followed by 5 m at 95°C using the Sensoquest lab cycler (Göttingen, Germany).

### 2.7 miRNome array

Prior to analyses, cDNA samples were diluted ten times. Expression of 1008 miRNAs plus six quality controls and six reference RNAs were quantified by qPCR using the 96 well plate Human miRNome miScript® miRNA PCR array (Qiagen) as described by the manufacturer (see Fig. 1). miRNAs with Ct values over 35 were classed as undetected. Raw data were normalized against the six reference RNAs and, as subsequent validation of miRNA expression by qPCR was normalized against only two reference RNAs, also against the selected two reference RNAs (*RNU‐6* and *SNORD68*) to check that these data were comparable with both normalization methods. Technical performance of the plates was checked using the integrated quality controls included on all the array plates.

### 2.8 Validation of miRNA expression by quantitative PCR

Quantification of the eight selected miRNAs for validation and two reference RNAs (*SNORD68* and *RNU‐6*) in individual samples was performed by qPCR using the Applied Biosystems® StepOnePlus™ system, the miScript SYBR Green PCR Kit and custom miScript primer assays (Qiagen, UK) as described by the manufacturer.

Prior to analysis of the qPCR data, the melt curves were checked for a single peak and a constant threshold value was set for each miRNA separately for all of the samples by taking the average of the set thresholds for each miRNA for every plate. Ct values greater than 35 were classed as undetected and raw data were normalized relative to the geometric mean of *RNU‐6* and *SNORD68*.

### 2.9 Statistical analyses

The data, expressed as adjusted relative copies (2^−ΔCt^ × 1000, relative to the geometric mean of *SNORD68* and *RNU‐6*), were analyzed using Minitab® v.17. Prior to analyses, data were checked for a normal distribution using the Kolmogorov‐Smirnov test. Data that were not normally‐distributed were transformed appropriately, using log_10_ or by taking the square root.

Differences between the two treatment groups were analyzed using the ANOVA General Linear Model (GLM), adjusting for pre‐intervention expression, age, gender, endoscopy procedure, smoking status, and BMI as covariates.

## 3 RESULTS

### 3.1 Participant characteristics

Samples from a total of 29 participants were utilized for this study, comprising 15 Control participants and 14 Double Intervention participants. Participant characteristics are summarized in Table 1.

**Table 1:** Participant characteristics for vontrol and RS + PD groups

Variable	Control	RS + PD	*P*‐value
N	15	14	
Age			
Mean (years)	49.2	51.1	0.669
Range (years)	30‐70	36‐74	
Gender			
Female (%)	7 (47)	3 (21)	0.256
Male (%)	8 (53)	11 (79)	
Ethnicity			
Caucasian (%)	14 (93)	14 (100)	
Black african (%)	1 (7)	0 (0)	
Endoscopy procedure			
Colonoscopy (%)	3 (20)	3 (21)	0.924
Flexible sigmoidoscopy (%)	12 (80)	11 (79)	
BMI			
Mean (kg/m^2^)	29.7	29.7	0.971
Range (kg/m^2^)	23.5‐37.0	23.5‐40.0	
Smoking status			
Never (%)	7 (46.6)	4 (28.6)	0.553
Former (%)	4 (26.7)	6 (42.8)	
Current (%)	4 (26.7)	4 (28.6)	

Mean age of participants in both groups was similar (49 and 51 years for Control and RS + PD, respectively), with the youngest participant being aged 30 and oldest aged 74. With the exception of one Control participant, all participants were Caucasian. In both groups, colonoscopy was the main endoscopy procedure at baseline. The participants were well‐matched for BMI (most were overweight or obese) and smoking behavior (73% and 71% were never or former smokers for Control and RS + PD groups, respectively). The observed similarity in participant characteristics is evidence of the success of the randomization process.

### 3.2 Human miRNome miScript miRNA PCR array

**Figure 2:**
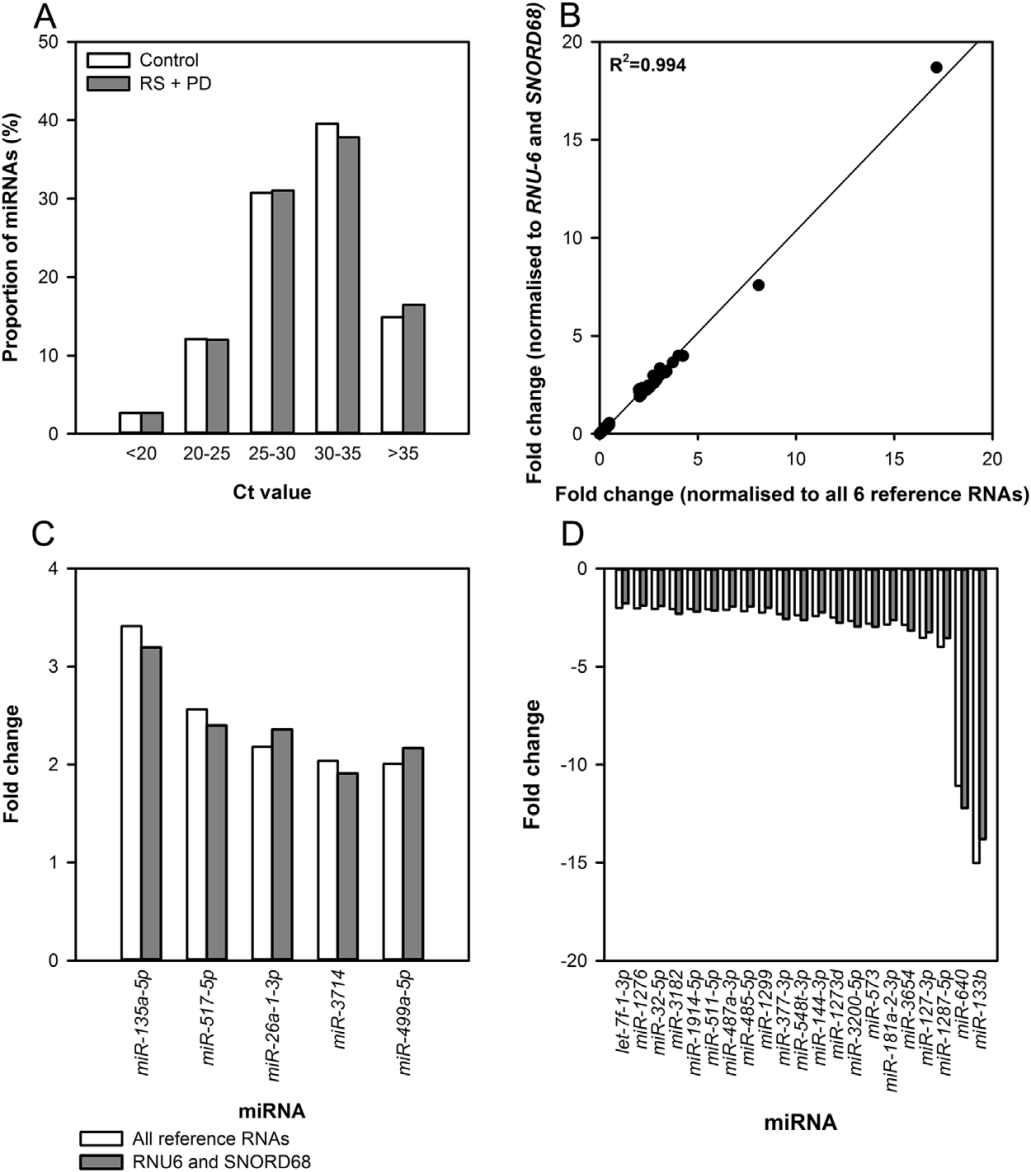
Whole miRNome PCR array (A) proportion of miRNAs with Ct values in different ranges, (B) correlation of data presented as fold‐change normalised relative to *RNU‐6* and *SNORD‐68* versus all six reference miRNAs, and miRNAs showing (C) ≥2‐fold up‐regulation and (D) ≥2‐fold down‐regulation in the RS + PD group compared with the Control group

The expression of 1008 miRNAs was quantified in pooled post‐intervention samples from participants in the Control and from the RS + PD group using Qiagen's Human miRNome array. All of the array plates passed the quality control checks. A total of 217 miRNAs (Ct values ≥35), equating to 22% of the 1008 quantified miRNAs, were undetected in one or both of the Control and RS + PD samples. The proportions of miRNAs in each Ct value range were comparable for both the Control and Intervention samples (Fig. 2A).

A cut‐off value of twofold difference (up‐ or down‐regulated) in expression, relative to all six reference RNAs, for the miRNAs between the RS + PD group and the Control group was used as an index of potentially differentially‐expressed miRNAs. A total of 33 miRNAs were up‐regulated ≥2‐fold and 78 miRNAs were down‐regulated ≥2‐fold in the Intervention compared with Control sample (Additional File 2).

### 3.3 Normalization of PCR array data

Raw data were normalized relative to all six reference RNAs included on the array plates and to the two selected reference RNAs (*RNU‐6* and *SNORD68)* used during the validation stage of the study. We checked that both normalization methods were comparable for all 111 miRNAs showing a fold‐change ≥ 2 (Fig. 2B). From the 1008 miRNAs for which there were probes on the array, 69 miRNAs showed ≥2‐fold difference in expression when normalized relative to only *RNU‐6* and *SNORD‐68* compared with 111 for normalization to all six reference RNAs. In all cases where the normalization methods resulted in different fold‐change results, fold‐changes were very small (between −1.13 and 1.12) and the difference in fold‐change between the two normalization methods did not exceed 0.14.

### 3.4 Selection of miRNAs for individual participant analysis

From the analysis of the 1008 miRNAs, two lists were compiled comprising miRNAs with ≥2‐fold up‐ or down‐regulation, which included a total of 111 miRNAs. Each list was then analyzed individually and those with a comment of “OKAY” or “A” on the miScript miRNA PCR Array Data Analysis Web Portal output were selected; this reduced the number of miRNAs to six up‐regulated ≥2‐fold and 23 down‐regulated ≥2‐fold. The miScript miRNA PCR Array Data Analysis Web Portal defined a comment of “OKAY” as a miRNA with reasonably low Ct values (<30) in both samples and comment of “A” as a miRNA with a relatively high (>30) Ct value in either the control or test sample and a reasonably low Ct value (<30) in the other sample. Because of inadequate melt curves, *miR‐3156‐5p, miR‐637*, and *miR‐600* were excluded at this stage, leaving five miRNAs up‐regulated ≥2‐fold (Fig. 2C) and 21 down‐regulated ≥2‐fold (Fig. 2D).

From the remaining 26 miRNAs, the top three miRNAs showing the greatest fold up‐regulation and the top four miRNAs showing the greatest fold down‐regulation were selected for further investigation. These were: *miR‐135a‐5p, miR‐517‐5p*, and *miR‐26a‐1‐3p* (upregulated genes) and *miR‐133b, miR‐640, miR‐1287‐5p*, and *miR‐127‐3p* (down‐regulated genes). In addition, *miR‐32* was selected due to its role in colorectal carcinogenesis.^17^

### 3.5 Quantification of miRNA expression in individual participants by qPCR

qPCR was run using samples from each individual participant in the Control group (*n* = 15) and in the RS + PD group (*n* = 14) for the eight selected miRNAs. Differences in post‐intervention miRNA expression were analyzed using the ANOVA GLM. Post‐intervention expression of *miR‐32* was approximately threefold greater in participants given RS and PD compared with the Control group (*P* = 0.033) (Table 2). There were no statistically significant differences in the expression of *miR‐26a, miR‐127, miR‐133b, miR‐135a, miR‐517, miR‐640*, or *miR‐1287* between the Control and RS + PD groups.

**Table 2:** Post‐intervention differences in miRNA expression in DISC Study participants allocated to the control group and to the RS + PD group

MiRNA	Control	RS + PD	*P*‐value
miR‐26a	0.916 (0.281‐1.000)	0.521 (0.521‐1.421)	0.343
miR‐32	0.564 (0.343‐0.926)	1.762* (0.980‐3.168)	0.033
miR‐127	7.550 (4.140‐10.960)	8.260 (4.497‐12.023)	0.806
miR‐133b	0.757 (0.452‐1.266)	1.054 (0.546‐2.038)	0.468
miR‐135a	2.963 (1.930‐4.549)	4.375 (2.774‐6.902)	0.285
miR‐517	0.888 (0.617‐1.207)	1.147 (0.749‐1.630)	0.372
miR‐640	0.429 (0.219‐0.742)	0.402 (0.173‐0.778)	0.902
miR‐1287	1.059 (0.411‐2.733)	1.560 (0.576‐4.228)	0.598

Data are expressed as adjusted copies. Data are presented as least square means (LSMs) and 95% confidence intervals (CIs) are included in parentheses.

*P < 0.05 (ANOVA GLM).

### 3.6 Comparison of PCR array and qPCR of individual participant data

Quantification of the eight selected miRNAs in individual samples allowed for comparison with the originally obtained PCR array data. For the PCR array data, fold‐change was calculated for each miRNA by dividing the relative copies (2^−ΔCt^) for the pooled RS + PD group sample by that for the pooled Control group sample. For the individual participant analysis stage, the means of the relative copies for each treatment group were calculated and from this the fold‐change was estimated. The resulting data are summarized in Table 3. The direction of fold‐change were the same for seven out of eight miRNAs, the exception being *miR‐133b*, and we observed some differences in the degree of change, for example the magnitude of fold‐change in *miR‐135a* for individual participant data was half that observed in the screening array (1.49 vs 3.19).

**Table 3:** Comparison of fold‐changes in miRNA expression between control and RS + PD samples using PCR array and subsequent qPCR validation on individual participant samples

MiRNA	Whole miRNome PCR array	qPCR validation	Same fold‐change direction
*miR‐135a*	3.19	1.49 (0.26)	Yes
*miR‐517*	2.40	1.73 (0.59)	Yes
*miR‐26a*	2.36	1.51 (0.32)	Yes
*miR‐32*	−1.91	−1.12 (0.31)	Yes
*miR‐127*	−3.24	−1.06 (0.17)	Yes
*miR‐1287*	−3.55	−1.05 (0.38)	Yes
*miR‐640*	−12.21	−1.12 (0.30)	Yes
*miR‐133b*	−13.79	1.20 (0.44)	No

Data are presented as fold‐changes (RS + PD group compared with the control group) where data were normalized relative to *RNU‐6* and *SNORD68*. Data for the qPCR validation were calculated using the mean of all individual samples. SEM are presented in parentheses.

## 4 DISCUSSION

miRNAs regulate the expression of thousands of genes by inhibiting their expression and, consequently, have effects on many biological processes including those involved in tumorigenesis.^7^, ^15^, ^18^ Abnormal expression of miRNAs is implicated in the pathogenesis of CRC, where it is associated with disease progression, treatment response, and prognosis.^19^, ^20^ miRNAs have potential to be used as cancer biomarkers since altered expression is observed not only in colorectal tissue but also in the plasma, stool, and urine from CRC patients.^11^, ^12^ However, little is known about the potential of miRNAs as early markers of CRC risk and how dietary factors may modulate these markers.

In the current study, we used a multi‐step approach to identify miRNAs in the colorectal mucosa of healthy individuals that are potentially regulated by two NDCs (RS and PD). Using a whole miRNome PCR array screen, we first compared the expression of 1008 miRNAs in pooled samples from rectal mucosal biopsies from participants who were randomized to RS + PD or to Control for 50 d. The use of pooling techniques has been applied widely in “omics” studies including those analyzing miRNA expression^21^, ^22^, ^23^ and is particularly valuable when samples are precious or limited.^24^ This screening stage revealed 33 miRNAs that were up‐regulated and 78 miRNAs that were down‐regulated by ≥2‐fold in the RS + PD group compared with the Control group. From these, eight miRNAs (*miR‐26a, miR‐21, miR‐133b, miR‐135a, miR‐517, miR‐640* and *miR‐1287*, and *miR‐32*) were selected for validation in individual participant samples. Validation by qPCR revealed consistent fold‐change direction for seven of the eight miRNAs. A possible explanation for the inconsistency observed for *miR‐133b* is the increased chance of error associated with the PCR array, whereas qPCR analysis of individual participant samples provides a more reliable and robust method with reduced chance of error, especially as all analyses were performed in duplicate. Fold‐changes were lower in the qPCR data compared with the array data and we expect that this resulted from differences in miRNA abundance in the pooled samples, which may not have been mathematically equivalent to the mean of the abundances in individual participant samples.

Post‐intervention, *miR‐32* expression was approximately threefold greater in participants supplemented with RS + PD compared with the Control (*P* = 0.033). At first sight, our findings are surprising as *miR‐32* expression has been found to be up‐regulated in colon tumor tissue compared with normal tissue and increased with advancing tumor stage.^25^ *miR‐32* has been shown to promote proliferation, migration, and invasion in colorectal and gastric cancer cells and reduces apoptosis.^17^, ^26^, ^27^ There also appears to be a connection between *miR‐32* and inflammation via the regulation of tumor necrosis factor receptor‐associated factor‐3 (*TRAF3*), an activator of the non‐canonical NF‐κB pathway.^28^ Therefore, downregulation of *TRAF3* by *miR‐32* could lead to increased inflammation and effects on other processes such as cell proliferation and cell death through stimulated NF‐κB signaling.^29^, ^30^, ^31^

While our findings are contradictory to evidence supporting butyrate's anti‐inflammatory properties, it is possible that effects on *miR‐32* expression and consequently on the NF‐κB non‐canonical pathway could be one of the mechanisms via which NDCs modulate processes such as cell proliferation and apoptosis.^5^ The very limited research on the effects of NDCs on miRNA expression in the large bowel mucosa suggests that RS and butyrate may reduce the expression of oncogenic miRNAs.^14^, ^15^ To our knowledge, our study is the first to report the effects of NDC supplementation on *miR‐32* expression in the human colorectal epithelium. The observed increase in *miR‐32* expression could be one of the mechanisms through which, in healthy colorectal cells, butyrate stimulates cell proliferation.^32^, ^33^, ^34^ In contrast, NDCs and butyrate reduce cell proliferation and promote apoptosis in CRC cells,^35^, ^36^, ^37^, ^38^, ^39^ referred to as the butyrate paradox,^40^ and the role of miRNAs in mediating these phenomena in both healthy and cancer tissue deserves further investigation.

Although, we did not observe significant effects of NDCs on expression of the additional seven miRNAs quantified, further investigation of possible effects of NDCs and other dietary factors on these miRNA in larger studies is warranted. We observed considerable inter‐individual variation in response to supplementation (evidenced by relatively large SEM values—Table 3) so that despite mean differences of up to 50%, these were not statistically significant. Furthermore, some of these miRNAs may have potential for development as biomarkers in investigations of the protective or detrimental effects of dietary factors, such as red meat and dietary fibre, on CRC risk. For example, *miR‐1287* 41 and *miR‐517* 42 are upregulated in CRC tissue and *miR‐133b* (which has been proposed to be a tumor suppressor) is downregulated in SW‐620 and HT‐29 colon cells.^43^ In addition, *miR‐135a* has been identified as a biomarker of CRC in serum^44^ and was downregulated by butyrate and trichostatin A, another HDACi, in LT97 colon adenoma cells. This effect was associated with reduced cell proliferation, suggesting that *miR‐135a* may be involved in butyrate‐mediated inhibition of cell proliferation in CRC cells.^8^

This study is the first to use a multi‐step approach to identify miRNAs in the colorectal mucosa of healthy individuals that are regulated by RS and PD. Participants in the study had been shown at endoscopy to have a normal colon and rectum and supplementation using a randomized double‐blind design ensured that observed effects are likely to be causal. This study has shown that *miR‐32* expression in the colorectal mucosa of healthy human participants increased substantially after supplementation with RS + PD for 50 d. Given the potential roles of this miRNA in regulation of the cell cycle, inflammation, and interactions with the gut microbiota, further studies are warranted to confirm our findings and to investigate the utility of *miR‐32* as a diet‐responsive biomarker of gut health. The findings from this RCT study, although relatively small, provide valuable evidence of causality which is free from confounding issues associated with observational studies, such as the subjectivity of self‐reported dietary data.

## Supporting information

Additional Supporting Information may be found online in the supporting information tab for this article.

Additional File 2.
