# Supplementary material for: Non‐digestible carbohydrates supplementation increases miR‐32 expression in the healthy human colorectal epithelium: A randomized controlled trial
Source: Mol Carcinog. 2017 May 9;56(9):2104–11. doi: 10.1002/mc.22666 (PMC5573932; doi:10.1002/mc.22666)
Supplement: Supplementary file 1 — Additional File 2. [file MC-56-2104-s001.pdf]

Additional File 2

Mature ID	Fold change relative to all reference genes	Fold change relative to RNU-6 and SNORD68	Comments
hsa-miR-551a	17.15	18.69	B
hsa-miR-676-5p	8.10	7.58	B
hsa-miR-4251	4.25	3.98	B
hsa-miR-3137	4.01	3.99	B
hsa-miR-621	3.73	3.65	B
hsa-miR-135a-5p	3.41	3.19	OKAY
hsa-miR-3155b	3.33	3.12	B
hsa-miR-488-3p	3.07	3.35	B
hsa-miR-647	3.03	2.96	B
hsa-miR-876-3p	2.90	2.76	B
hsa-miR-3074-3p	2.77	2.59	B
hsa-miR-758-3p	2.73	2.98	B
hsa-miR-517-5p	2.56	2.40	A
hsa-miR-3140-3p	2.51	2.39	B
hsa-miR-3156-5p	2.51	2.35	A
hsa-miR-1914-3p	2.49	2.33	B
hsa-miR-3144-5p	2.49	2.47	B
hsa-miR-875-3p	2.45	2.29	B
hsa-miR-4285	2.42	2.41	B
hsa-miR-936	2.41	2.29	B
hsa-miR-2116-3p	2.39	2.24	B
hsa-miR-3605-3p	2.39	2.37	B
hsa-miR-31-3p	2.37	2.26	B
hsa-miR-541-3p	2.36	2.24	B
hsa-miR-26a-1-3p	2.18	2.36	A
hsa-miR-23a-5p	2.18	2.36	B
hsa-miR-4290	2.12	1.99	B
hsa-miR-3661	2.12	1.99	B
hsa-miR-3158-3p	2.10	1.99	B
hsa-miR-3714	2.04	1.91	A
hsa-miR-125b-1-3p	2.02	2.28	B
hsa-miR-499a-5p	2.01	2.17	A
hsa-miR-411-3p	2.00	2.26	B

Mature ID	Fold change relative to all reference genes	Fold change relative to RNU-6 and SNORD68	Comments
hsa-miR-637	-484.32	-517.18	OKAY
hsa-miR-600	-27.66	-25.38	A
hsa-miR-133b	-15.03	-13.79	OKAY
hsa-miR-640	-11.08	-12.21	A
hsa-miR-1537-3p	-6.56	-6.89	B
hsa-miR-206	-6.41	-5.88	B
hsa-miR-3117-3p	-6.19	-6.52	B
hsa-miR-516b-5p	-5.42	-4.80	B
hsa-miR-512-3p	-4.79	-4.39	B
hsa-miR-130a-5p	-4.56	-4.80	B
hsa-miR-1287-5p	-3.99	-3.55	OKAY
hsa-miR-1205	-3.99	-3.55	B
hsa-miR-4326	-3.81	-4.20	B
hsa-miR-509-5p	-3.78	-3.34	B
hsa-miR-105-5p	-3.73	-3.98	B
hsa-miR-127-3p	-3.53	-3.24	OKAY
hsa-miR-1323	-3.49	-3.11	B
hsa-miR-373-3p	-3.39	-3.62	B
hsa-miR-135b-3p	-3.35	-2.98	B
hsa-miR-449b-5p	-3.28	-2.92	B
hsa-miR-643	-3.27	-3.35	B
hsa-miR-3186-3p	-3.25	-3.58	B
hsa-miR-4304	-3.20	-3.53	B
hsa-miR-383-5p	-3.18	-3.40	B
hsa-miR-1911-3p	-3.12	-3.28	B
hsa-miR-3143	-2.99	-3.15	B
hsa-miR-3918	-2.93	-3.23	B
hsa-miR-3654	-2.87	-3.16	A
hsa-miR-181a-2-3p	-2.84	-2.63	A
hsa-miR-548b-3p	-2.83	-2.89	B
hsa-miR-573	-2.82	-2.96	OKAY
hsa-miR-619-3p	-2.79	-2.56	B
hsa-miR-630	-2.77	-2.54	B
hsa-miR-2115-5p	-2.72	-2.52	B
hsa-miR-509-3p	-2.70	-2.72	B
hsa-miR-3200-5p	-2.67	-2.95	OKAY
hsa-miR-767-5p	-2.60	-2.78	B
hsa-miR-518f-5p	-2.51	-2.68	B
hsa-miR-873-5p	-2.51	-2.68	B
hsa-miR-1273d	-2.50	-2.75	A
hsa-miR-524-3p	-2.44	-2.24	B

hsa-miR-144-3p	-2.43	-2.24	OKAY
hsa-miR-3939	-2.40	-2.52	B
hsa-miR-548t-3p	-2.38	-2.62	A
hsa-miR-4261	-2.38	-2.62	B
hsa-miR-412-3p	-2.36	-2.17	B
hsa-miR-211-5p	-2.33	-2.38	B
hsa-miR-377-3p	-2.33	-2.57	A
hsa-miR-4257	-2.31	-2.43	B
hsa-miR-4253	-2.31	-2.43	B
hsa-miR-639	-2.30	-2.11	B
hsa-miR-1266-5p	-2.26	-2.09	B
hsa-miR-34b-3p	-2.26	-2.01	B
hsa-miR-1299	-2.24	-2.00	A
hsa-miR-297	-2.23	-1.98	B
hsa-miR-3617-5p	-2.21	-2.36	B
hsa-miR-3917	-2.20	-2.32	B
hsa-miR-3142	-2.20	-2.32	B
hsa-miR-668-3p	-2.19	-2.30	B
hsa-miR-323b-5p	-2.18	-2.02	B
hsa-miR-485-5p	-2.18	-1.94	A
hsa-miR-3692-3p	-2.14	-2.26	B
hsa-miR-1262	-2.14	-1.98	B
hsa-miR-3922-3p	-2.13	-2.24	B
hsa-miR-623	-2.11	-2.26	B
hsa-miR-487a-3p	-2.11	-1.94	A
hsa-miR-582-3p	-2.10	-2.25	B
hsa-miR-3605-5p	-2.10	-2.21	B
hsa-miR-3160-3p	-2.09	-2.10	B
hsa-miR-511-5p	-2.09	-2.13	OKAY
hsa-miR-1914-5p	-2.09	-2.19	A
hsa-miR-3182	-2.08	-2.30	A
hsa-miR-32-5p	-2.07	-1.91	OKAY
hsa-miR-302c-3p	-2.06	-1.90	B
hsa-miR-380-5p	-2.05	-1.82	B
hsa-miR-1276	-2.04	-1.88	A
hsa-miR-514a-3p	-2.02	-2.06	B
hsa-let-7f-1-3p	-2.01	-1.78	OKAY
